# Research on Boundary Displacement of Probe Trajectory Considering Deviations in Five-Axis Sweep Scanning Measurement

**DOI:** 10.3390/mi16070759

**Published:** 2025-06-27

**Authors:** Peng Chen, Tao Fang, Zhiyong Chang, Bowen Xue, Neng Wan

**Affiliations:** 1 Department of Mechanical Engineering, Northwestern Polytechnical University, Xi’an 710072, China; elijahchern@mail.nwpu.edu.cn (P.C.); fangtnwpu@mail.nwpu.edu.cn (T.F.); xbw_rhic@mail.nwpu.edu.cn (B.X.); wanneng@nwpu.edu.cn (N.W.); 2 Institute for Aero-Engine Smart Assembly of Shaanxi Province, Xi’an 710072, China

**Keywords:** five-axis sweep scanning measurement, deviations, constrained sector effect, boundary displacement

## Abstract

Five-axis sweep scanning measurement technology, as a novel contact measurement technology, offers excellent reachability and high measurement efficiency for complex parts. However, deviations between the measurement instructions based on the model and the workpiece exist, leading to mismatches between the intended and actual sweep scanning areas, which manifest as displacements of the scanning boundaries and subsequently impact the acquisition of sampling points. When these sampling points are utilized to evaluate the machining quality of workpieces, the accuracy and reliability of the assessment results are compromised. Therefore, by focusing on the phenomenon of boundary displacement in a five-axis sweep scanning measurement, the sampling principle has been analyzed, the constrained sector for the probe tip trajectory in a five-axis scanning measurement has been defined, and the concept of the trajectory constrained sector effect has been proposed for the first time. The constrained sector effect reveals how deviations affect the scanning boundary positions and acquisition of sampling points. Based on the constrained sector effect, the influence of deviations on boundary displacement and sampling point acquisition in single-patch and multiple-patch measurement scenarios is discussed. Furthermore, practical engineering recommendations are provided, aiming to reduce the impact of deviations on the completeness of sampling point acquisition.

## 1. Introduction

**Figure 1:**
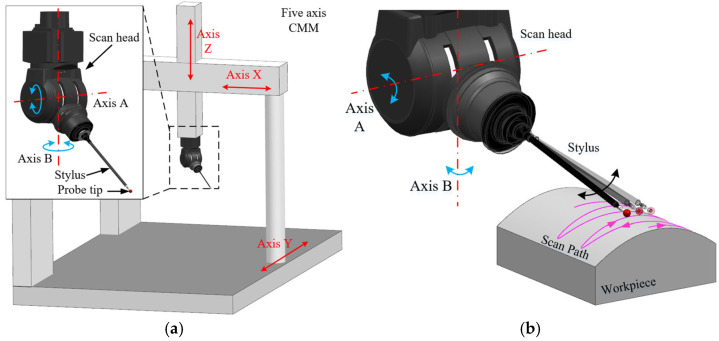
Five-axis sweep scanning measurement. (**a**) Five-axis CMM; (**b**) five-axis sweep scanning.

Complex parts characterized by free-form surfaces have increasingly emerged as fundamental components in contemporary industrial design and manufacturing. With their intricate structures and excellent functional performance, these parts demonstrate broad application potential in aerospace, automotive, energy, and other fields [1,2,3], promoting both product performance and industrial innovation. However, it is precisely these complex features that make their inspection highly challenging [4,5]. Coordinate measuring machines (CMMs) are widely used in industry due to their high accuracy and robustness [6]. Yet, as the demand for measuring increasingly complex surfaces grows, traditional three-axis or “3+1” axis CMMs often fall short in efficiency and accessibility [7]. To address these challenges, five-axis coordinate measurements—where all five axes move simultaneously—are increasingly recognized and utilized [8,9]. Compared with traditional three-axis systems, a five-axis measurement allows the stylus to move along the X, Y, and Z directions while rotating around the A and B axes, as shown in Figure 1a. Driven by the scan head, the stylus performs continuous and rapid scanning over the workpiece surface, referred to as a five-axis sweep scanning measurement, as shown in Figure 1b. During the five-axis sweep scanning, a lightweight stylus is driven by the scan head to perform rapid and continuous surface measurements at speeds of up to 500 mm/s. Meanwhile, the heavy frame requires minimal movement, with the stylus executing the majority of measurement motions [10]. This measurement technique eliminates the frequent back-and-forth movements of the stylus in traditional point-by-point measurements and minimizes the dynamic errors resulting from the heavy frame’s frequent acceleration and deceleration during measurements. Consequently, this method significantly enhances measurement efficiency without compromising accuracy [11].

A five-axis sweep scanning measurement facilitates the inspection of complex parts due to its high flexibility and speed. In recent years, several scholars have conducted in-depth research on the path planning of five-axis sweep scanning measurements. For example, Zhang [12] proposed a strategy for automatically generating sweep scanning paths for free-form surfaces, which significantly improved inspection efficiency compared to the commonly used isoplanar zigzag method. To maximize the overall surface sweep rate (SSR), Chen [13] devised a spiral strip algorithm for generating five-axis sweep scanning paths, comprehensively accounting for probe trajectory smoothness and collision-free conditions, and ultimately validated its feasibility through experiments. Hu [14] proposed a five-axis sweep scanning strategy for the automatic planning of barrel surfaces, which eliminated surface partitions through optimized path planning, thereby reducing the non-scanning time and improving inspection efficiency. Shen [15] combined the image algorithms with B-spline fitting algorithms to devise a smooth and interference-free scanning path generation algorithm, ensuring the smoothness and non-interference of the trajectory. Zhang [16,17] conducted comprehensive research on the five-axis scanning method for porous skeleton parts, resolving the conflict between continuous measurement and efficient inspection due to the topological complexity of skeleton curves. The aforementioned studies provide various five-axis sweep scanning path planning strategies, not only improving inspection efficiency but also inspiring novel ideas for the application of this measurement technique across a broader range of components.

**Figure 2:**
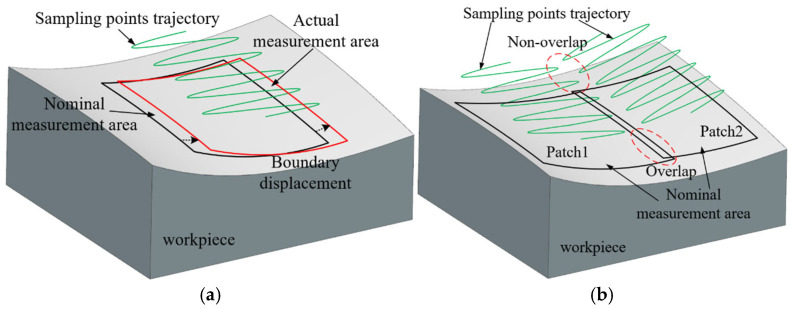
The impact of boundary displacement on sampling in a five-axis sweep scanning measurement. (**a**) Boundary displacement of the measurement area; (**b**) non-overlapping sampling points on adjacent patches.

Traditional sampling methods, such as point-by-point or single-line scanning, can only acquire limited data during each pass. In contrast, five-axis sweep scanning samples dense and continuous curves across the target surface, rapidly collecting a large number of data points. The fundamental difference lies in transitioning from sampling one-dimensional curves to full three-dimensional surface areas. In practice, path planning for five-axis sweep scanning typically follows a theoretical model. However, in practical applications, deviations between the actual part and the theoretical measurement model—caused by surface profile errors or coordinate system misalignments—can lead to the displacement of the sampling area, a phenomenon referred to as boundary displacement, as shown in Figure 2a. When measuring complex parts such as blades or impellers, surface segmentation is often required during path planning to avoid interference [18,19]. These segments are measured separately, and boundary displacement may result in non-overlapping sampling points between adjacent patches, as shown in Figure 2b. Here, the sampling points refer to the uncompensated points obtained directly. When the non-overlapping sampling points are used to evaluate the geometric tolerance of the workpiece, such as for a surface profile evaluation or curve profile evaluation, it may affect the accuracy and reliability of the evaluation results.

Five-axis sweep scanning measurement technology is a novel technology, with current research primarily focusing on non-interference path planning. However, the impact of surface deviations on the measurement area and sampling accuracy remains largely unexplored. In practice, many engineering cases have shown that the overlap between the paths planned for adjacent patches significantly affects sampling completeness. Since the overlap length is often determined based on the programmer’s experience, too short an overlap may leave gaps between adjacent patches, while an overly long overlap can compromise path planning efficiency and increase the risk of probe interference. Therefore, studying the influence of deviations on the five-axis sweep scanning motion and sampling is of great significance for harnessing the high-precision and efficient measurement advantages of five-axis sweep scanning measurement technology, and for promoting its widespread adoption and application.

This study aims to solve the problems such as measurement interruption and the insufficient coverage of sampling points caused by the boundary displacement phenomenon resulting from the deviation between the measurement instructions and the workpiece in the five-axis sweep scanning measurement, thereby improving the integrity and accuracy of complex surface measurements. A novel concept, referred to as the constrained sector effect of the probe trajectory, is proposed to reveal the intrinsic mechanism by which the trajectory remains confined within a specific spatial region, despite the presence of various types of deviations. Furthermore, based on this effect, the influence of boundary displacement on sampling continuity and evaluation accuracy is analyzed in both single-patch and multi-patch measurement scenarios.

To comprehensively explore the aforementioned issues, the structure of this paper is organized as follows. In Section 2, we analyze the sampling principle of a five-axis sweep scanning measurement and define the constrained sector. In Section 3, considering the deviations between the measurement instructions and the workpiece, the adjustment and control principle of the five-axis CMM on the stylus is analyzed, and the constrained sector effect of the sampling trajectory during five-axis sweep scanning is proposed for the first time. In Section 4, the boundary displacement in the single-patch and multiple-patch scenarios is analyzed and discussed based on the constrained sector effect. In Section 5, two typical experiments are used to verify the correctness of the constrained sector effect and the influence of boundary displacement on the acquisition of sampling points. The conclusion is presented in Section 6.

## 2. Constrained Sector of the Probe Tip Trajectory Based on Sampling Principle in Five-Axis Sweep Scanning

With the development of science and technology, the sampling method of contact measurements has evolved from traditional point-by-point sampling to curve scanning, and then to the current surface sweep scanning, namely five-axis sweep scanning. However, different sampling methods have different sampling principles [20], especially for five-axis sweep scanning.

### 2.1. Sampling Principle of Five-Axis Sweep Scanning Measurement

#### 2.1.1. Acquisition of Sampling Points

**Figure 3:**
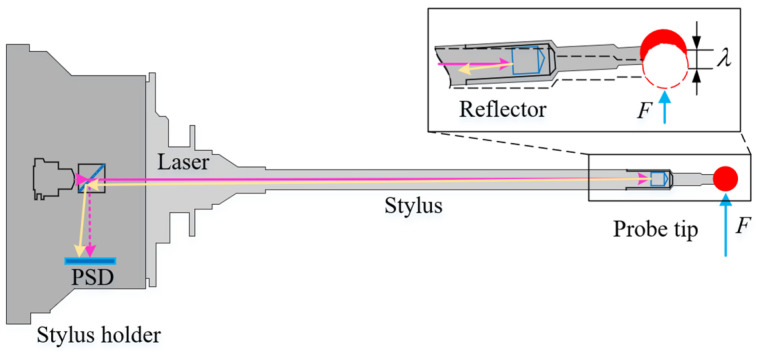
Acquisition sampling points in five-axis sweep scanning measurement.

A traditional three-axis CMM typically uses touch trigger probes to sample points [21,22]. During sampling, when the contact force between the probe and the surface reaches a predetermined threshold, a signal is triggered that causes the machine to capture the measurement point information. In contrast to a traditional three-axis CMM, the scanning probe in five-axis sweep scanning uses laser and stylus deformation to obtain the real-time position of the probe tip center [23], as shown in Figure 3. When a force F is applied to the stylus by the measured part, the probe tip undergoes a slight deflection λ from its unforced position (indicated by the red dash circle). This deflection causes a change in the positional information of the laser spot detected by the position-sensitive detector (PSD), in accordance with the principles of light reflection. Consequently, the real-time position of the probe tip center can be deduced from the laser spot position. Because of its high sensitivity and exceptional stability [24,25], the scanning probe captures minute surface variations and reduces pre-travel errors. To maintain accuracy and stability, it is imperative to keep the deflection of the stylus within an acceptable range during measurements. For example, the REVO-RSP2 of the Renishaw Inc. scanning probe achieves standard sampling when the deflection λ remains within the range of 0.03 mm to 0.2 mm.

Therefore, to acquire accurate sampling points, the stylus must maintain continuous contact with the workpiece while keeping probe deformation within a predefined range. Additionally, to perform continuous surface scanning, the five-axis CMM must drive the stylus to execute a sweep scanning motion across the surface.

#### 2.1.2. Driving Method of the Stylus

A traditional three-axis CMM typically samples the workpiece either by collecting individual sampling points or through curve scanning. However, a five-axis CMM drives the stylus to execute continuous and rapid scanning sampling. The different sampling methods result in significant differences between the path planning and the driving method of the stylus in the five-axis measurement compared to the three-axis measurement.

**Figure 4:**
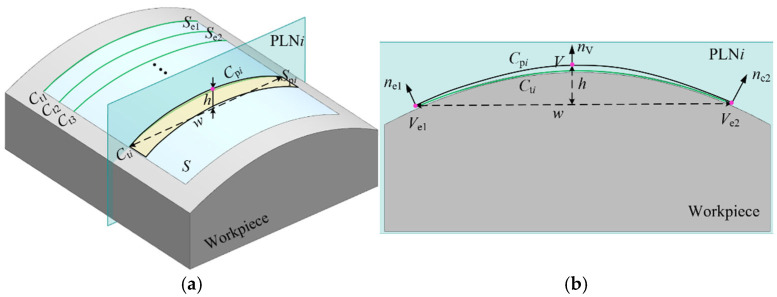
Approximate expression of free-form surface. (**a**) Approximate expression of surface; (**b**) approximation of the intersection curve by a parabola.

Before conducting a five-axis sweep scanning measurement, it is necessary to input the surface information into the five-axis CMM. Given the complexity of the mathematical expression of the free-form surface, the surface to be measured is typically approximated by multiple simpler surfaces in most instances. As shown in Figure 4a, for a given surface S, multiple segment planes (PLNs) can be defined to divide it into several surface segments $S_{e}$. Each segment can be represented by the intersection curves formed between adjacent planes. Based on the Weierstrass approximation theorem, the intersection curve $C_{t}$ can be uniformly approximated using a polynomial. Due to the ease of acquiring its parameters, the parabola $C_{p}$ is frequently used to approximate the intersection curve $C_{t}$. As shown in Figure 4b, the parabola $C_{p}$ is represented by the vertex V, the vertex normal $n_{V}$, the width w, and the height h. Therefore, the surface S is approximated by multiple parabolas.

(1) $$\begin{array}{l} C_{pi}\left(V_{i},n_{Vi},w_{i},h_{i},\mathrm{PLN}i\right),i=1,2,3,\ldots ,n\\ S\approx \left\{S_{pi}\left(C_{pi},C_{pi+1}\right)\right\},i=1,2,3,\ldots ,n-1 \end{array}$$

where $C_{pi}$ is the parabola, V is the vertex of the parabola, w and h are the width and height of the parabola, $S_{pi}$ is the ruled surface, and n is the number of parabolas.

According to the international standard ISO22093-2011(DIMS) [26], measurement instructions are expressed in the way of defining waypoints or defining path curves. In general, the measurement path of the surface is planned by defining multiple curves in five-axis sweep scanning. The specific description is as follows:

$$F\left(\mathrm{lname}\right)=PATH/SURFACE,PTDATA,x,y,z,i,j,k,w,h,\mathrm{PCS},angz1,angy2,angz3,VAR.$$

where PATH and SURFACE are keywords in the definition statement. PTDATA is a keyword followed by the cross-section data of the surface to be measured; x,y,z,i,j,k are the position and normal of the center line of the scanned surface at which the width and height parameters are applied, and w,h are the width and height of the scan at the cross-section, respectively. PCS is a keyword followed by angz1,angy2,angz3, which represent the orientation of the stylus at the specified position in the part coordinate system. *VAR* represents a variable that can continue to be information in PTDATA. These measurement instructions not only represent the surface information to be measured but also the basis for the five-axis CMM to assign drive to each axis.

**Figure 5:**
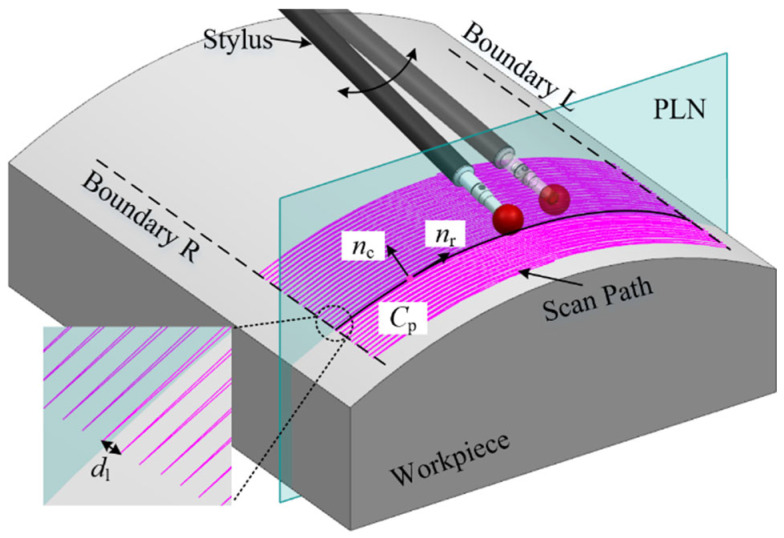
Driving method of five-axis sweep scanning.

According to the motion characteristics of five-axis sweep scanning, the stylus undergoes a reciprocating periodic motion across the workpiece surface while being driven by the scan head, as shown in Figure 5. A complete cycle involves the time taken by the stylus to move from boundary R to boundary L and then back to boundary R. During this cycle, the distance that the stylus is pulled back by the scan head is known as the line spacing $d_{l}$. Generally, the line spacing is relatively small, ranging from approximately 0.05 mm to 0.5 mm. It can be approximately assumed that within half a cycle, the trajectory of the probe tip lies on the segmented plane PLN. On plane PLN, the force exerted by the five-axis CMM on the stylus is decomposed into two components: the tangential and normal directions of the surface at the point along the parabola. The tangential direction is considered the driving vector $n_{r}$, guiding the continuous sweep scanning motion. The normal direction serves as the deflection control vector $n_{c}$, facilitating the application of force necessary for the stylus to maintain contact with the workpiece and undergo deformation [27]. The endpoints of the parabola establish the boundaries of the sweep scanning motion. For clarity, the relationship between the sweep scanning trajectory and the deviations is analyzed in the segmented plane PLN.

### 2.2. Definition of Constrained Sector

**Figure 6:**
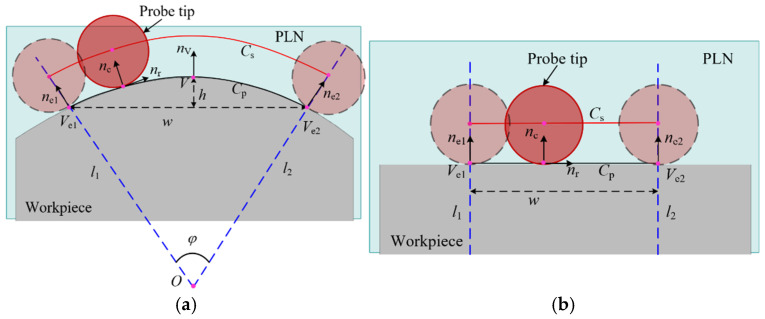
The constrained sector of the sampling trajectory in five-axis sweep scanning. (**a**) The constraint lines are not parallel; (**b**) the constraint lines are parallel.

In general, measurement instructions are obtained according to the model. Represented by the parabola $C_{p}$, these instructions control the sweep scanning motion of the stylus across the workpiece surface. The trajectory of the contact points between the stylus and the workpiece surface is denoted as $C_{c}$. The center point of the probe tip is acquired by the five-axis CMM, also known as the sampling point. The trajectory formed by sampling points is denoted as $C_{s}$. These trajectories are the physical manifestations of the measurement instructions in the physical space. When profile deviations and parabola-fitting deviations are disregarded, the contact point trajectory $C_{c}$ coincides with the parabola $C_{p}$ in the segmented plane PLN, and the sampling trajectory $C_{s}$ serves as an offset curve of the contact point trajectory $C_{c}$, with the offset distance being the probe tip radius r, as shown in Figure 6. The boundaries of the sampling trajectory are determined by two lines, $l_{1}$ and $l_{2}$, which pass through the end of the parabola $V_{e1}$ and $V_{e2}$, and are parallel to the normal $n_{e1}$ and $n_{e2}$ at the end, respectively. The lines $l_{1}$ and $l_{2}$ constrain the motion of the probe tip within a range bounded by these two lines. Consequently, the lines $l_{1}$ and $l_{2}$ are referred to as the constraint lines for the sampling trajectory. When the constraint lines $l_{1}$ and $l_{2}$ are not parallel, they intersect at a point O, forming the angle known as the constraint angle φ. The sector area surrounded by the constraint lines and the point O is called the constrained sector of the sampling trajectory, simply referred to as the constrained sector. The sampling trajectory is constrained by the constrained sector, as shown in Figure 6a. When the constraint lines are parallel, the constraint angle φ=0, and the region formed by the two constraint lines is a special case of the constrained sector, as shown in Figure 6b.

In theory, the trajectory of sampling points is constrained by the constrained sector. However, in practical measurements, the trajectory of sampling points is not solely determined by the measurement instructions but is also influenced by the actual profile of the workpiece. The impact of deviations on sampling will be explored in the subsequent chapter.

## 3. Constrained Sector Effect on Probe Tip Trajectory Considering Deviations

It is well known that there are machining deviations between the workpiece and its nominal model. A machining deviation refers to the distance between the theoretical profile and the actual profile along the surface normal direction. Moreover, a coordinate system deviation arises between the measurement coordinate system, which is established during the measurement process, and the model coordinate system. Both the machining deviation and the coordinate system deviation are independent of the measurement instructions and are uniformly regarded as the profile deviation that exists between the workpiece and the model. In addition, the measurement instructions are obtained based on the model. According to the acquisition of the measurement instructions, there are inevitably fitting deviations between the measurement instructions and the model. Profile deviations or fitting deviations are regarded as deviations between the measurement instructions and the workpiece. These deviations ultimately manifest as discrepancies between the actual sampling point trajectory and the measurement instructions, thereby influencing the actual measurement area.

**Figure 7:**
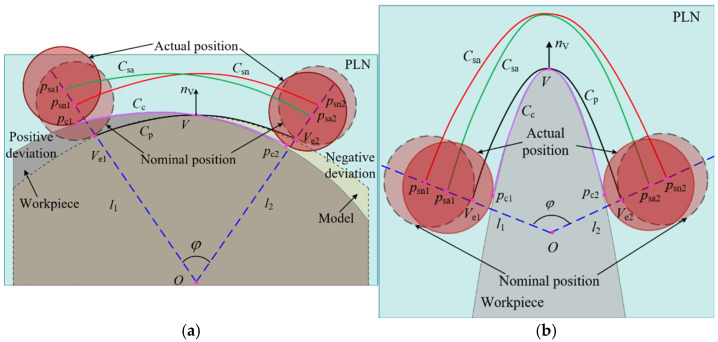
The constrained sector effect in five-axis sweep scanning. (**a**) Profile deviation only; (**b**) fitting deviation only.

The purpose of a measurement is to obtain the surface information of the workpiece. When deviations arise between the measurement instructions and the actual workpiece, the five-axis CMM needs to adjust the stylus along the deflection control vector to maintain contact with the workpiece, thereby fulfilling the objective of sampling. Simultaneously, it also needs to drive the stylus to perform periodic movements on the surface of the workpiece. There are cases where fitting deviations are neglected and only profile deviations are present, as shown in Figure 7a. At the endpoints of the parabola, the profile deviations are either positive or negative. The stylus is driven to execute a sweep scanning motion based on the parabola $C_{p}$. According to the constrained sector, the theoretical sampling trajectory is $C_{\mathrm{sn}}$. However, due to the presence of profile deviations, the five-axis CMM not only needs to adapt to the deviations to ensure that the stylus completes the sampling, but it also adheres to the constraints imposed by the measurement instructions, sampling within the specified region. Therefore, when the stylus theoretically moves to the point $p_{\mathrm{sn}1}$ according to the measurement instructions, the existence of a positive profile deviation prompts the five-axis CMM to adjust the motion of the probe along the deflection control vector $n_{c}$. Given that the boundary line specified by the measurement instructions for the probe’s motion is $l_{1}$, the actual stylus is constrained to move solely to the point $p_{\mathrm{sa}1}$, where the contact point between the probe tip and the workpiece is $p_{c1}$.

Similarly, when the stylus is theoretically directed to move to $p_{\mathrm{sn}2}$, the motion of the stylus is adjusted by the five-axis CMM in the opposite direction of the deflection due to the negative profile deviation. The actual stylus position is relocated to point $p_{\mathrm{sa}2}$, with the contact point being $p_{c2}$. Therefore, when there are deviations between the measurement instructions and the workpiece, the behavior of the five-axis CMM adjusting the motion of the stylus based on the actual shape of the workpiece to achieve sampling is termed as the adjustment and control of the five-axis CMM, referred to as regulation. The existence of this regulation ensures that the five-axis CMM does not rigidly follow the pre-planned path, in contrast to the operation of a five-axis CNC machining machine, thereby enhancing its adaptability and accuracy in measuring complex geometries. The actual contact point trajectory of the probe tip with the workpiece is denoted as $C_{c}$, while the actual sampling trajectory of the probe, which serves as an offset curve of $C_{c}$, is represented by $C_{\mathrm{sa}}$. In scenarios where deviations exist between the measurement instructions and the workpiece, based on the regulation and constrained sector, it can be known that the boundary points of the actual sampling point trajectory $C_{\mathrm{sa}}$ lie on the constraint lines $l_{1}$ and $l_{2}$. And the $C_{\mathrm{sa}}$ remains consistently within the confines of the constrained sector. This phenomenon is called the constrained sector effect of the five-axis sweep scanning measurement trajectory, abbreviated as the “Constrained Sector Effect”. The constrained sector effect reveals the influence of deviations on the motion trajectory of the stylus.

When a profile deviation is ignored, only a fitting deviation exists, as shown in Figure 7b. The actual sampling trajectory $C_{\mathrm{sa}}$ remains constrained within the constrained sector by the regulation of the five-axis CMM, thereby satisfying the constrained sector effect. It is evident that regardless of whether the deviations are attributed to a profile deviation or fitting deviation, the motion trajectory of the probe tip consistently adheres to the constrained sector effect. However, the phenomenon that the actual contact point trajectory $C_{c}$ caused by the deviation is different from the parabolic $C_{p}$, which affects the boundary of the actual sweep area, resulting in the displacement of the sweep boundary. This displacement impacts the acquisition of sampling points and can even influence the evaluation of the machining quality of the workpiece, which is described in detail in the subsequent chapter.

## 4. Sweep Scanning Boundary Displacement Based on the Constrained Sector Effect

Boundary displacement reflects the difference between the actual measurement area and the theoretical measurement area, which affects the acquisition of sampling points. Sampling points constitute an essential basis for evaluating the quality of workpieces. When the acquisition of sampling points is impacted by boundary displacement, the evaluation of workpiece quality, which is predicated on these points, may suffer from a loss of due accuracy and reliability.

### 4.1. Boundary Displacement in a Single Patch

**Figure 8:**
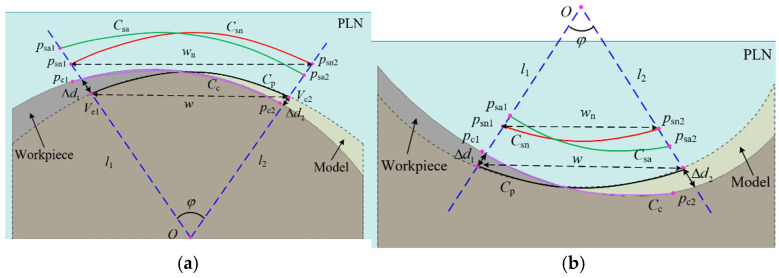
The relationship between profile deviation and boundary displacement. (**a**) Convex surface; (**b**) concave surface.

When measuring a single patch, the projection length of the sampling trajectory along the width direction of the parabola is defined as the sampling width, which represents the coverage of the area to be measured by the sampling points. In Section 3, it can be seen that the profile deviation of the workpiece and the fitting deviation of the parabola ultimately manifest as a deviation between the measurement instructions and the workpiece. For the convenience of description, the deviations described in the following section all refer to the deviations between the measurement instructions and the workpiece. As shown in Figure 8a, there is a positive deviation $\Delta d_{1}$ and a negative deviation $\Delta d_{2}$ at both ends of the scanning boundary, respectively. The boundary points of the theoretical sampling trajectory are points $p_{sn1}$ and $p_{sn2}$. According to the constrained sector effect, the boundary points of the actual sampling trajectory are points $p_{sa1}$ and $p_{sa2}$, respectively. Consequently, a positive deviation causes the sampling trajectory to exceed the theoretical sweep boundaries in the direction of the parabolic width, whereas a negative deviation leads to the sampling trajectory being confined within a smaller area than the theoretical sweep boundaries. In this scenario, the boundaries of the actual sampling area undergo a displacement. The actual contact point trajectory is $C_{c}$. Since the five-axis CMM directly acquires sampling points, the contact points usually need to be derived through compensation. Consequently, when the boundaries of the sampling trajectory undergo displacement, the boundaries of the contact point trajectory also shift accordingly, with the boundary points identified as $p_{c1}$ and $p_{c2}$.

Similarly, if the workpiece to be measured is a concave surface, as shown in Figure 8b, a positive deviation leads to a smaller actual sampling width, while a negative deviation leads to a larger actual sampling width. However, whether it is a convex surface or a concave surface, a positive deviation causes the actual contact point boundary to exceed the theoretical contact point boundary, while a negative deviation causes the actual contact point boundary to be smaller than the theoretical contact point boundary.

**Figure 9:**
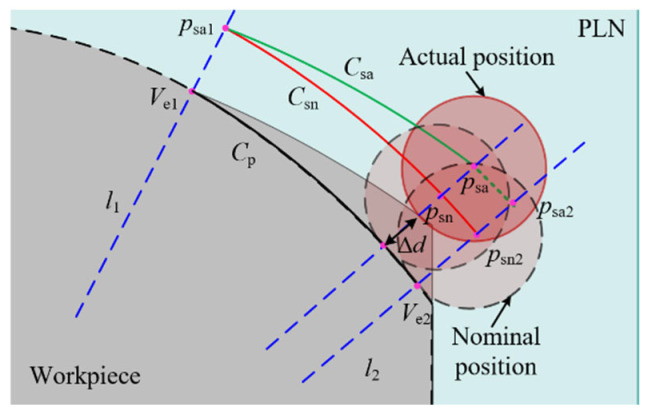
Measurement interruption caused by boundary displacement.

Deviations lead to alterations in the sampling width, subsequently causing displacements in the boundaries of the sampling area. The boundary displacement of the sampling area provides a reasonable explanation for the frequently observed inconsistencies between the actual and theoretical scanning paths in practice, particularly when the probe tip slips outside the intended measurement region during the five-axis sweep scanning. As shown in Figure 9, when the sampling area is near the edge of a workpiece with a positive deviation, the theoretical motion trajectory of the probe tip is $C_{sn}$, with $p_{sn2}$ as one of the boundary points. However, the actual motion trajectory of the probe tip is $C_{sa}$. When the probe tip reaches the point $p_{sa}$, it has already touched the edge of the workpiece. Nevertheless, due to the constrained sector effect, the stylus continues to move forward until it reaches the constraint line $l_{2}$, at which point the probe tip may slip off the surface of the workpiece, potentially leading to measurement interruption and negatively impacting both sampling accuracy and measurement efficiency.

### 4.2. Boundary Displacement in Multiple Patches

**Figure 10:**
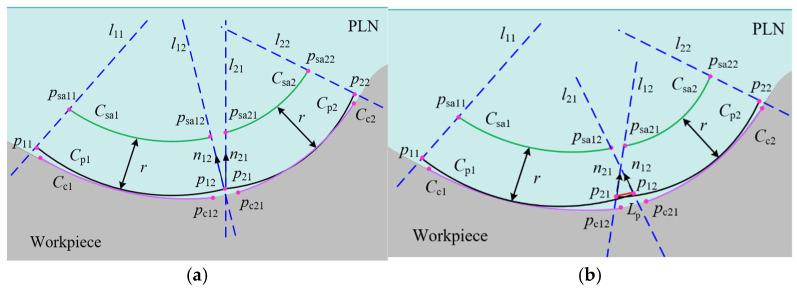
The relation of parabolas between adjacent patches. (**a**) Zero-order continuity of adjacent parabolas; (**b**) overlap between adjacent parabolas.

When the surface under measurement is segmented into multiple patches, it is possible for the endpoints $p_{12}$ and $p_{21}$ of the parabolas $C_{p1}$ and $C_{p2}$, belonging to adjacent patches, respectively, to coincide, as shown in Figure 10a. Alternatively, the endpoints may not coincide but overlap slightly, as shown in Figure 10b. Despite the continuity or overlap of the parabolas across adjacent patches, deviations result in the non-coincidence of the normal vectors $n_{12}$ and $n_{21}$ at the respective endpoints. According to the constrained sector effect, the endpoints $p_{sa12}$ and $p_{sa21}$ of the sampling trajectories $C_{sa1}$ and $C_{sa2}$, respectively, also do not overlap. This results in a non-overlapping of sampling points across adjacent patches, and potentially, the actual contact point trajectories $C_{c1}$ and $C_{c2}$ are also non-overlapping.

In addition, even if the parabolas of adjacent patches overlap, the overlapping length is approximated by the distance $L_{p}$ between the endpoints $p_{12}$ and $p_{21}$. According to the constrained sector effect, it is important to note that a small overlap length or significant deviation still results in the non-overlapping adjacent sampling trajectories $C_{sa1}$ and $C_{sa2}$. Therefore, in multiple-patch scanning, the displacement of the scanning boundary caused by deviation factors affects the acquisition of sampling points, resulting in the phenomenon of non-overlapping sampling points between adjacent patches. There are no sampling points between adjacent patches, which means that the contact points at that position cannot be obtained through compensation. When non-overlapping sampling points are used for evaluating the profile of a workpiece, it affects the accuracy and reliability of the evaluation. Therefore, it is crucial to ensure overlap between the sampling points of adjacent patches during scanning.

**Figure 11:**
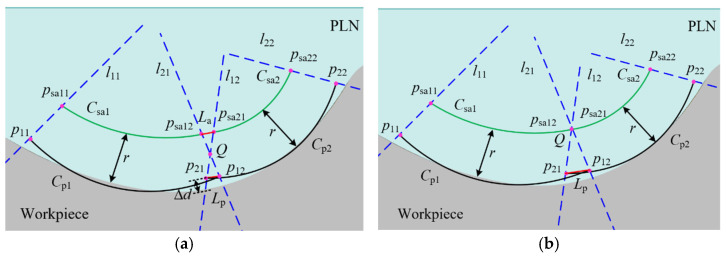
Calculation of the non-overlap length. (**a**) Calculation of the non-overlap length of sampling points; (**b**) calculation of the minimum overlap length of adjacent parabolas.

As shown in Figure 11a, there exists an overlap between the parabolas of the adjacent patches $C_{p1}$ and $C_{p2}$. The normal vectors at the endpoints $p_{12}$ and $p_{21}$ are denoted as $n_{12}$ and $n_{21}$, respectively. The overlap length of the parabolas is as follows:

(2) $$L_{p}=\left|p_{21}-p_{12}\right|$$

There is a deviation, denoted as Δd, between the parabola and the workpiece. Specifically, Δd<0 represents a negative deviation, while Δd>0 indicates a positive deviation. The normal vectors $n_{12}$ and $n_{21}$ do not coincide. Based on the constrained sector effect, it is inferred that the sampling trajectories $C_{sa1}$ and $C_{sa2}$ are not overlapping, with a non-overlapping length of $L_{a}$. The constraint lines $l_{12}$ and $l_{21}$, which pass through the endpoints $p_{12}$ and $p_{21}$, are defined as follows:

(3) $$\begin{array}{l} l_{12}:y-y_{p_{12}}=k_{12}\left(x-x_{p_{12}}\right)\\ l_{21}:y-y_{p_{21}}=k_{21}\left(x-x_{p_{21}}\right) \end{array}$$

The line $l_{p_{12}p_{21}}$ passing through the endpoints $p_{12}$ and $p_{21}$ can be expressed as follows:

(4) $$l_{p_{12}p_{21}}:y-y_{p_{12}}=\frac{y_{p_{12}}-y_{p_{21}}}{x_{p_{12}}-x_{p_{21}}}\left(x-x_{p_{12}}\right)$$

where $x_{p_{12}}$, $y_{p_{12}}$, $x_{p_{21}}$, $y_{p_{21}}$ are the coordinates of point $p_{12}$ and point $p_{21}$. $k_{12}$, $k_{21}$ are calculated by the normal vectors $n_{12}$ and $n_{21}$.

When $k_{12}=k_{21}$, the two constraint lines are parallel and have no intersection point. When $k_{12}\neq k_{21}$, there is an intersection point Q between the two constraint lines, which can be represented as follows:

(5) $$Q=\left(\frac{y_{p_{21}}-y_{p_{12}}+k_{12}x_{p_{12}}-k_{21}x_{p_{21}}}{k_{12}-k_{21}},\frac{k_{12}y_{p_{21}}-k_{21}y_{p_{12}}-k_{12}k_{21}\left(x_{p_{21}}-x_{p_{12}}\right)}{k_{12}-k_{21}}\right)$$

The distance D from Q to line $l_{p_{12}p_{21}}$ is expressed as follows:

(6) $$D=\frac{\left|k_{p}x_{Q}-y_{Q}+b_{p}\right|}{\sqrt{{k_{p}}^{2}+1}}$$

where $k_{p}$ and $b_{p}$ are the slop and the y-intercept of the line $l_{p_{12}p_{21}}$, respectively.

If D≥r+Δd, there exists an overlap among the sampling points between adjacent patches. Conversely, if D<r+Δd, the sampling points between adjacent patches are not overlapping. The non-overlapping length $L_{a}$ of the sampling points is derived from the similarity of triangles as follows:

(7) $$L_{a}=\frac{L_{p}\left(r+\Delta d-D\right)}{D}$$

where *r* is the probe tip radius, and $L_{p}$ is the overlap length of the parabolas.

As can be seen from the above analysis, the overlapping of the sampling points between adjacent patches is related not only to the deviation between the measurement instructions and the workpiece but also to the probe tip radius. Reducing either the fitting deviation or the probe tip radius facilitates the overlap of sampling points. When the fitting deviation remains constant, increasing the overlap length of the parabolas increases the distance D, thereby reducing the non-overlapping length of the sampling points. Assuming a uniform deviation between the measurement instructions and the workpiece at adjacent patch positions, the angle between the two constraint lines $l_{12}$ and $l_{21}$ typically does not exceed 40°. The distances from the intersection point Q to the endpoints $p_{12}$ and $p_{21}$ can be approximately considered equal. As shown in Figure 11b, to ensure the overlap of adjacent sampling trajectories, the intersection point Q must at least lie on the sampling trajectory. In this case, the minimum overlap length $L_{p}$ of the two parabolas is as follows:

(8) $$L_{p}=2\sin20{}^{\circ}\left(r+\Delta d\right)\approx 0.684\left(r+\Delta d\right)$$

The above analysis reveals the influence of deviations on sampling point acquisition. To ensure overlapping sampling between adjacent patches during multiple-patch measurements and to not affect the workpiece quality evaluation, three feasible engineering suggestions are provided:

**(1) Minimize fitting deviations**. Deviation is an important factor that causes boundary displacement and affects the acquisition of sampling points. A machining deviation of a workpiece is inevitable, but a fitting deviation can be controlled. For instance, a large measurement area can be subdivided into smaller segments, thereby reducing the width or height of the fitted parabolic segments. This subdivision improves fitting accuracy by minimizing the deviation within each local region. In addition, using higher-order curves with smaller fitting deviations to approximate the surface under measurement facilitates the overlap of sampling points.

**(2) Reduce the probe tip radius**. The size of the probe tip is related to the contact point trajectory. The smaller the probe tip radius used, the more likely it is that the sampling points overlap.

**(3) Increase the overlap length between adjacent parabolas**. Although increasing the overlap length may compromise the fitting accuracy of the parabolas, it is a direct, effective, and easy method of promoting an overlap of the sampling trajectories. To ensure overlapping sampling points, the overlap length between parabolas should be at least 0.68 times or more than the sum of the probe tip radius and the deviation.

## 5. Experiments

To validate the correctness of the constrained sector effect and the influence of boundary displacement on sampling trajectory, five-axis sweep scanning experiments were conducted on a standard sphere and an aero-engine blade, respectively. The equipment used in the experiment was the Renishaw Agility five-axis CMM, with a maximum permissible error of 1.9 μm. The software used for measurement was Modus 1.11.

### 5.1. Five-Axis Sweep Scanning Measurement Experiments on a Standard Sphere

**Figure 12:**
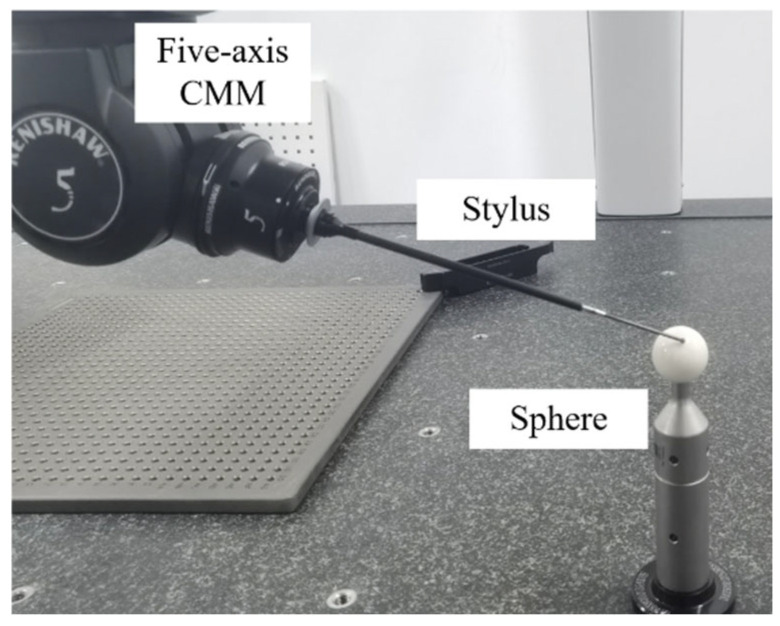
Five-axis sweep scanning measurement standard sphere experiment.

**Figure 13:**
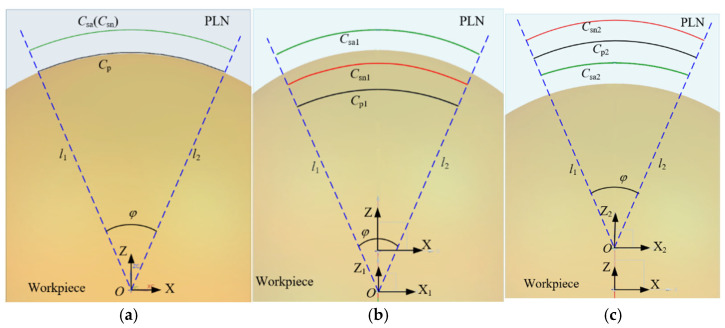
Measurement coordinate system translation along the Z-axis. (**a**) Translation of 0 mm. (**b**) Translation of −3 mm. (**c**) Translation of 3 mm.

**Figure 14:**
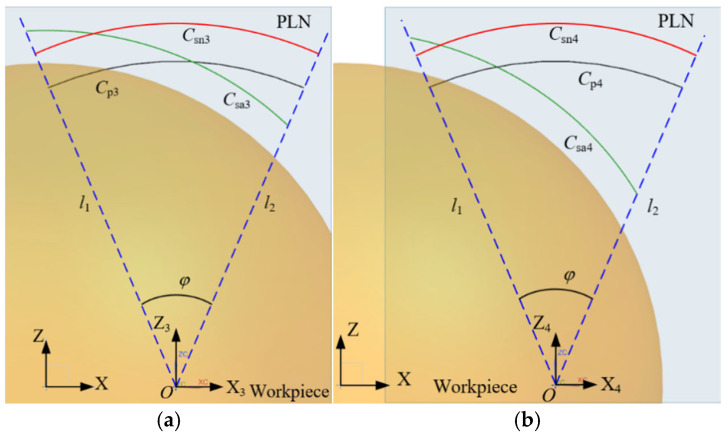
Measurement coordinate system translation along the X-axis. (**a**) Translation of 6 mm; (**b**) translation of 10 mm.

A standard sphere was selected as the experimental object because of its high machining accuracy, which can be approximated as having no profile deviation. The fitting deviation of the parabola is also small and negligible when a five-axis sweep scanning path is planned for a sphere. The top region of a 30 mm diameter standard sphere was selected, with a parabola width of *w* = 12 mm and height of *h* = 1.2 mm. The sweep scanning speed was set to 10 mm/s, employing the D3 × L50 mm stylus, as shown in Figure 12. Profile deviations were artificially created using a translating coordinate system in the experiments. Two sets of experiments were designed. The first set involved translating the measurement coordinate system along the Z-axis by 0 mm, −3 mm, and 3 mm, while the workpiece remained stationary. This setup simulates the presence of only positive or negative profile deviations in the workpiece. The second set involved translating the measurement coordinate system along the X-axis by 6 mm and 10 mm, while the workpiece remained stationary. This setup simulates the presence of positive and negative profile deviations at the two boundary positions of the workpiece. In both sets of experiments, the sweep scanning measurement programs were the same. The trajectory of the probe tip center during one scanning period was projected onto the PLN, with the experimental results shown in Figure 13 and Figure 14.

As shown in Figure 13a, when there is no profile deviation between the measurement instructions and the actual profile of the workpiece, the actual sampling trajectory $C_{\mathrm{sa}}$ coincides with the theoretical trajectory $C_{\mathrm{sn}}$. However, with only positive profile deviation in the workpiece, as shown in Figure 13b, the stylus must actively adjust to maintain contact with the workpiece. In this case, the five-axis measurement system automatically adjusts the probe’s orientation based on the shape of the workpiece, resulting in a change in the probe’s direction to adapt to the profile. Although the sampling trajectory changes spatially during this process, it remains within the preset constrained sector region, with its boundary limited by the constraint line. Similarly, with only a negative profile deviation in the workpiece, as shown in Figure 13c, the stylus automatically adjusts its orientation based on the direction of the deviation to maintain contact with the workpiece. However, the sampling trajectory still strictly stays within the constrained sector region. This behavior further confirms the presence of the constrained sector effect, whose core feature is that the probe’s motion range is always restricted within a sector-shaped space determined by both the geometric boundary of the measurement instructions and the regulation strategy of the measurement system.

Furthermore, with both positive and negative profile deviations in the workpiece, as shown in Figure 14a,b, the relative positional relationship between the measurement instructions and the workpiece profile becomes more complex. Despite this complexity, the sampling trajectory remains confined within the constrained sector, and there is no occurrence of sampling beyond the constrained lines. This phenomenon demonstrates that the constrained sector effect is not only applicable to single-directional deviations but is a universal motion rule for the probe.

In summary, the constrained sector effect is essentially a probe motion law jointly determined by the geometric constraints of the measurement instructions and multiple factors such as obstacle avoidance logic and the regulation of the measurement system. From the changes in the sampling trajectory caused by positive and negative deviations to the changes in trajectory shape under mixed deviations, the experiments fully validate the existence of the constrained sector effect, as well as its adaptability and manifestation under different deviation conditions. This effect not only reveals the relationship between the probe motion and the workpiece deviation in the five-axis sweep scanning measurement but also provides a theoretical basis for the subsequent optimization of five-axis sweep scanning measurement strategies.

### 5.2. Five-Axis Sweep Scanning Measurement Experiments on a Blade

**Figure 15:**
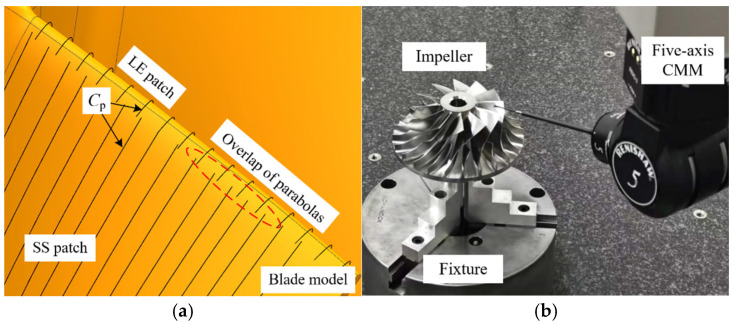
Five-axis sweep scanning measurement blade experiment. (**a**) Acquisition of measurement instructions. (**b**) The experimental site of blade sweep scanning measurement.

**Figure 16:**
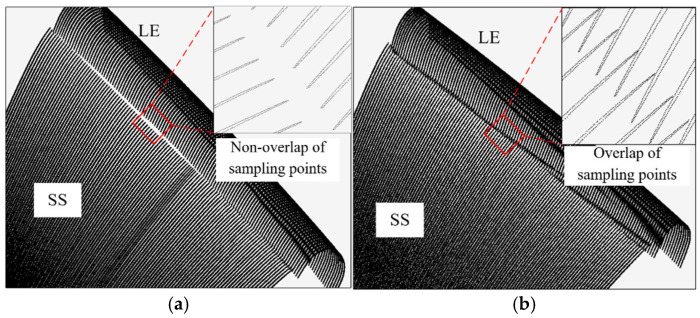
Obtained sampling points of the blade. (**a**) Non-overlap of sampling points obtained; (**b**) overlap of sampling points obtained.

The full surface measurement of complex parts represented by blades usually requires splitting the blade surface into multiple patches during path planning. Using a certain blade as an example, the surface to be measured was segmented into a leading edge (LE) patch, a trailing edge (TE) patch, a pressure side (PS) patch, and a suction side (SS) patch. During parabolic fitting, the intentional overlap between patches is considered and established, as shown in Figure 15a. Two sets of experiments were conducted, each with different overlap lengths of the parabolas. The five-axis CMM equipped with the stylus D3 × L50 mm was used to conduct a sweep scanning measurement of the blade, as shown in Figure 15b. For instance, Table 1 displays the parabolic information of the patches and the overlap of sampling points, focusing on the PLN at the 9th and 18th parabolic lines of the leading edge. The actual values refer to both the programmed overlap length of the parabolas and the actual overlap length of the obtained sampling points. The predicted values represent the overlap length of the parabolas and the overlap length of the sampling points, both calculated based on the boundary displacement method outlined in Section 4.2. The sampling points obtained by the two sets of experiments are shown in Figure 16.

In PLN9, the actual overlap length of the parabolas between the LE patch and the SS patch is 0.38 mm, and the obtained sampling points are non-overlapping, forming a gap between the two patches, with a non-overlap length of about 0.8 mm, as shown in Figure 16a. The predicted overlap length of the parabolas is 0.403 mm, and the sampling points do not overlap, with a non-overlap length of 0.76 mm, which is consistent with the experiment. In PLN18, the actual overlap length of the parabolas between the LE and the PS patches is 0.41 mm, with the sampling points showing overlap, where the actual overlap length is 0.12 mm. The predicted overlap length of the parabolas is 0.44 mm, while the overlap length of the sampling points is 0.16 mm.

To ensure that overlap between the sampling points occurs, the required overlapping length can be calculated using Equation (8). For example, in PLN9, the minimum required parabolic overlapping length was calculated to be no less than 1.05 mm. When the overlapping length was increased to 1.05 mm, the model predicted the existence of overlap, with an overlapping length of 0.23 mm. The actual measured overlapping length was 0.24 mm, as shown in Figure 16b, confirming the prediction. Similarly, in cross-section PLN18, the measured overlapping length between the LE patch and the SS patch was 1.16 mm. The model predicted the occurrence of overlap, with an overlapping length of 0.69 mm. The actual measurement showed an overlapping length of 0.75 mm. These results demonstrate the model’s ability to predict the occurrence and extent of overlap. Therefore, the model can be used not only to validate overlap in existing designs but also to optimize the overlapping length of the parabolic patch during programming. This ensures the presence of sampling point overlap and supports complete sampling coverage, which is essential for an accurate profile evaluation.

**Figure 17:**
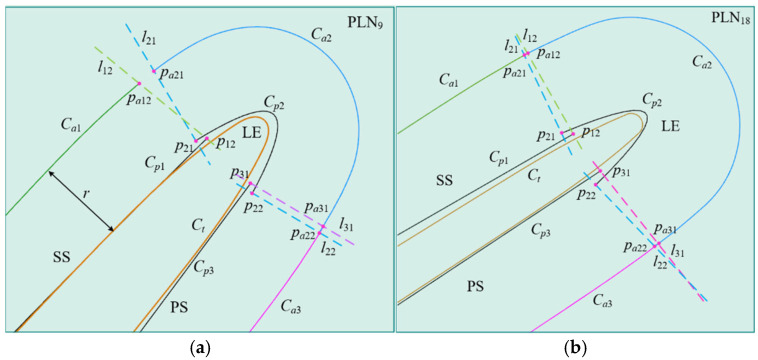
Boundary displacement caused by fitting deviation. (**a**) Trajectory curves in PLN9; (**b**) trajectory curves in PLN18.

Figure 17 displays the nominal blade profile curve, the actual probe tip trajectory, and the parabolic information for PLN9 and PLN18. Although there are deviations between the measurement instructions and the workpiece profile, including fitting and machining deviations, the sampling trajectories in all regions still adhere to the constrained sector effect. Whether in the SS and PS area with smaller deviations or in the LE area with larger deviations, the actual sampling trajectories never exceed the boundary of the constraint line defined by the measurement instructions. This further demonstrates the universality of the constrained sector effect. No matter how the local geometric characteristics of the workpiece profile change, and regardless of the type and magnitude of the deviation, the measurement system can always limit the movement range of the probe within a specific sector space through the regulation mechanism. This not only reflects the adaptability of the measurement system to the deviation working conditions, but also indicates that the constrained sector effect can provide a general sampling behavior law for the five-axis measurement task of complex surfaces.

In addition, as shown in Figure 17a, the fitting deviation of the LE is more pronounced when compared to the SS patch and PS patch. Although there is an overlap between the parabolas $C_{p1}$ and $C_{p2}$, there exists a notable disparity in the direction vectors of the constraint lines $l_{12}$ and $l_{21}$. According to the constrained sector effect, the actual positions of the probe tip are $p_{sa12}$ and $p_{sa21}$, which leads to non-overlapping sampling points between the LE patch and the SS patch. Conversely, as shown in Figure 17b, the disparity in the direction vectors between the constraint lines $l_{12}$ and $l_{21}$ is comparatively minor for the LE patch and the SS patch. When there is an overlap between the parabolas, the obtained sampling points also overlap.

**Figure 18:**
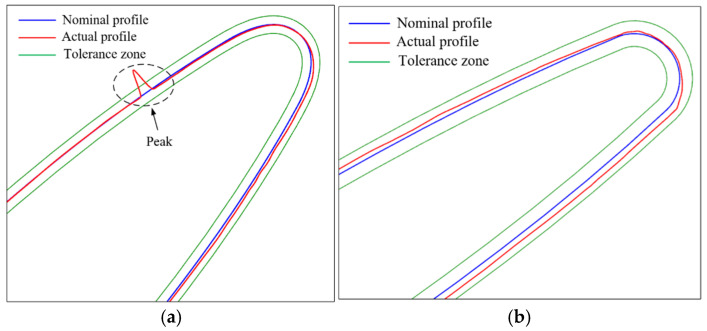
Blade profile evaluation using non-overlapping sampling points. (**a**) Profile curve in PLN9; (**b**) profile curve in PLN18.

**Figure 19:**
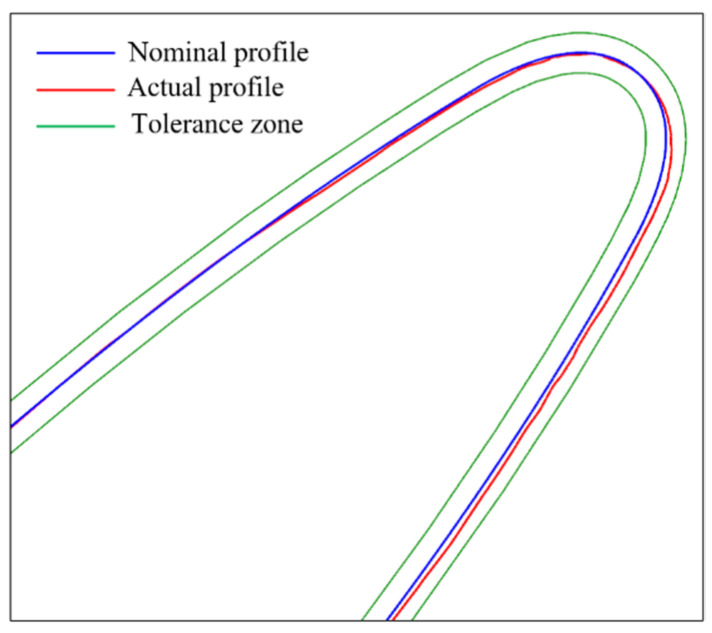
Blade profile evaluation using overlapping sampling points.

The sampling points require compensation before being used for quality evaluation. The sampling points obtained from the two experiments were imported into the commercial software Modus to analyze and evaluate the blade profile at sections PLN9 and PLN18. A tolerance zone of ±0.05 mm was established. The evaluation results are shown in Figure 18. During the evaluation of the blade profile at section PLN9 with non-overlapping sampling points, a “peak” emerged at the transition location from the SS pitch to the LE pitch, as shown in Figure 18a. At this specific location, the profile exceeded the tolerance zone by 0.095 mm, whereas the profiles at other locations remained within the tolerance limits. An examination of the actual workpiece surface showed no signs of chipping or burrs. No abnormal conditions were found in the profile evaluation at section PLN18, as shown in Figure 18b, and the evaluation results were within acceptable limits. However, when the profile at section PLN9 is evaluated again using the overlapping sampling points, as shown in Figure 19, the “peak” in the profile vanishes, rendering the blade profile acceptable. Numerous engineering examples have demonstrated that utilizing non-overlapping sampling points with the commercial software Modus for a workpiece quality evaluation may result in inaccurate outcomes due to missing data between adjacent patches or issues with compensation algorithms, thereby compromising the credibility of the blade profile evaluation.

## 6. Conclusions

This paper focuses on the phenomenon of non-overlapping sampling points between adjacent patches in five-axis sweep scanning. The sampling principle of five-axis sweep scanning is analyzed, and the constrained sector of a sweep scanning trajectory is defined. Considering the deviation between the measurement instructions and the workpiece, and based on the regulation of the stylus by the five-axis CMM, the constrained sector effect of the probe tip trajectory is proposed. The influence of boundary displacement caused by the constrained sector effect on sampling point acquisition in single-patch and multi-patch measurements is analyzed. The constrained sector effect reveals the relationship between stylus motion and the measurement instructions when deviations occur. At the same time, a reasonable explanation is given for the phenomenon where the sampling points of adjacent patches are non-overlapping. Based on theoretical analysis and experiments, it can be found that boundary displacement is inevitable when the five-axis sweep scanning method is used to measure complex parts. However, the influence of boundary displacement on sampling and part quality evaluations can be reduced in the following ways: (1) minimize fitting deviations; (2) reduce the probe tip radius; (3) increase the overlap between adjacent patches. In general, to ensure the overlap of sampling points, the overlap length of the parabolas should be at least 0.68 times the sum of the probe tip radius and profile deviation.

However, this study still has certain limitations. Firstly, it only focuses on analyzing probe tip motion when the measurement instructions are defined as path curves, neglecting other potential forms of measurement instructions. Secondly, the relationship between deviation and adjustment capability is not explained in detail. Therefore, these issues will also be the focus of our future research.

## Figures and Tables

**Table 1:** Parabola information and overlap length of sampling points (mm).

		Endpoint of Parabola		Normal Vector of Endpoint		Overlap Length of Sampling Points (*L*_a_)	
		*p*_12_	*p*_21_	*n*_12_	*n*_21_	Actual	Predicted
Test1	PLN9	(−2.01, −1.62)	(−2.43, −1.67)	(−0.72, 0.54)	(−0.36, 0.93)	non-overlap	non-overlap
	PLN18	(−3.48, −1.75)	(−3.89, −1.90)	(−0.54, 0.84)	(−0.38, 0.92)	0.12	0.16
Test2	PLN9	(−1.86, −1.37)	(−2.66, −1.84)	(−0.76, 0.64)	(−0.41, 0.91)	0.24	0.23
	PLN18	(−3.22, −1.64)	(−3.94, −2.31)	(−0.55, 0.83)	(−0.39, 0.92)	0.75	0.69

## Data Availability

The original contributions presented in this study are included in the article. Further inquiries can be directed to the corresponding author(s).
